# Music Activities and Mental Health Recovery: Service Users’ Perspectives Presented in the CHIME Framework

**DOI:** 10.3390/ijerph18126638

**Published:** 2021-06-21

**Authors:** Janne Brammer Damsgaard, Anita Jensen

**Affiliations:** 1 Research Unit of Nursing and Healthcare, Institute of Public Health, Aarhus University, 8000 Aarhus, Denmark; 2 Center for Primary Health Care Research, Region Skåne, 205 02 Malmö, Sweden; anita.jensen@skane.se; 3 Department of Communication and Psychology, Aalborg University, 2450 Copenhagen, Denmark

**Keywords:** mental health recovery, CHIME, music activities, service users’ perspectives

## Abstract

Internationally, mental health service developments are increasingly informed by the principles of recovery, and the availability of arts and creative activities are becoming more common as part of provision. Mental health service users’ experiences, reflecting on the complex nature of using music participation in recovery are, however, limited. This essay considers literature that explores how music can support mental health service users in a recovery process. We have selected studies that include a broad spectrum of music activities, as well as literature considering various concepts about recovery. The conceptual recovery framework CHIME, that includes five important components in the recovery process, is used as the backdrop for exploring music activities as a contribution to recovery-oriented practice and services in mental health care. Eleven key components are identified in which music can support the recovery process: Feelings of equality; Social and emotional wellbeing; Tolerance; Hope and social agency; Triggering encounters; Redefining and reframing; A social practice; Moments of flow and peak experiences; Moments of meaning; Continuity; and Potentials instead of limitations. This essay concludes that the experiential knowledge of music activities from service users’ perspectives is essential knowledge when developing and using music activities in mental health recovery services. While this essay acknowledges that music activities can also produce unintended negative outcomes, the focus is on the positive contributions of music to mental health recovery processes.

## 1. Introduction

There is an increase in research that explores the potential benefits of music, participation in music activities, and music therapy [1,2,3] in mental health care contexts. Many professions are involved in researching the relationship between music activities, i.e., music education, music therapy, community music, music medicine, and everyday uses of music [4], and wider health parameters. This includes professions such as: music therapists, psychologists, nurses, neurologists, teachers, occupational therapists, medical doctors, and architects; hence, many disciplines are involved in researching the connections between music, health, and wellbeing.

We all experience music in unique ways [5], which may remind mental health care professionals of the importance of tuning into the service users’ valuable expertise about their experience of music activities. Internationally, we see mental health services being informed by the principles of recovery [5], allowing the experiences and perspectives of service users to come to the forefront.

The scope of different approaches to music activities is broad; however, within this essay we do not distinguish between these approaches but focus on the service users’ experiences with music activities. We consider music activities to include active music making (singing, playing an instrument, song writing), music medicine (see Bonde’s Music in hospitals. Systematic overview of agents and interventions) [6], as well as music activities used in music therapy. While the use of music in health and wellbeing is experiencing an increased interest, music therapy has a long history of research that dates back to the beginning of the 20th century [7]. Hence, the profession of music therapy has been developing research and practice for many years [8] and has had an important impact on how music activities can be understood as a tool for social interaction in different clinical and non-clinical settings [6].

Within the field of music therapy, we see an increasing amount of research about clinical effects, different client populations [9,10,11,12], and clinical recovery through the alleviation of symptoms. Such studies are based on expert interventions with diagnostic criteria [13] and provide evidence-based practice by using data that primarily focuses on outcome measures [14].

This is important in documenting the use of music therapy as an effective clinical treatment in mental health service provision. However, according to McCaffrey and Solli [15,16], a one-sided focus on outcome measures can neglect the perspectives of service users and their valuable information about their personal experiences [17]. This is a point of discussion not only from the perspective of participation in music activities [18] but also in their perspectives of mental health services generally. This shows that we are moving away from a clinical understanding of care towards a more recovery-focused service provision, where inclusion of service users’ perspectives is crucial [19,20]. Empowering service users is, however, a slow and transformative process [19]. As recovery-oriented services are emerging, a more holistic approach to developing evidence is needed with a focus on gathering valuable experiential knowledge to inform service delivery [21,22,23].

We understand that there are also negative effects of music, including unhealthy psychological habits such as ‘rumination’ or overindulgence in ‘nostalgia’ or attraction to “(sad) music as manifestations of maladaptive mood regulation strategies” [24] (p. 147). Furthermore, music that is too loud, is played repetitively, or is politically offensive has been used as torture in concentration camps [25]. Music and noise can be seen as a collection of vibrational actions which may have influence on the mind and the body, which can stimulate beneficial or harmful bodily effects and provoke psychological reactions [26].

This essay aims to provide an insight into research, based on experiential knowledge of music activities and service users’ perspectives and relating to a variety of mental health disorders—we are not considering details of how the various activities are most appropriate for individual diagnosis. Furthermore, the essay will consider how the findings can strengthen or support existing understanding of practices in mental health services. We will introduce “personal and social recovery” as a user-oriented perspective on mental illness and mental health care. To emphasize the focus on personal and social recovery processes, the CHIME framework developed by Leamy and colleagues will be applied [27]. The framework contributes to an understanding of the stages and processes of recovery, providing a structure in which findings about music activities can be positioned. We have also sought inspiration from other authors [28,29]. The CHIME framework is based on five core recovery elements: “Connectedness, Hope & Optimism, Identity, Meaning in life and Empowerment” [27] (p. 448). From the literature we intend to identify (a) important aspects (potentials) where music supports recovery processes, by focusing on service users’ experiences of participation and, (b) how such experiences may influence recovery-oriented practice and services in mental health care.

## 2. Recovery

WHO defines mental health as “A state of well-being in which the individual realizes his or her own abilities, can cope with the normal stresses of life, can work productively and fruitfully, and is able to make a contribution to his or her community” [30]. According to Slade, however, a distinction between mental illness and mental health is important, since mental disorders directly impact personal identity and the ability to maintain social roles [31]. To Slade, mental health must support both the reduction of mental illness and the improvement of mental health. Within the context of the COVID-19 pandemic, where significant changes to our daily lives and fear and worries are likely responses to perceived or real threats, the focus on mental health has become even more relevant.

In the context of civil rights and independent living movements, the concept of recovery was developed in the 1960s and the 1970s. This was a response to a history of expert-led treatment of mental illness that had led to stigmatization and deindividuation [32]. From this perspective, “the understanding of mental health recovery grew as a liberating movement and academic field to help people diagnosed with a mental illness reclaim their right to a safe, dignified and personal and gratifying life in their community despite his or her psychiatric condition” [33] (p. 11). The most frequently used definition of recovery was written by Anthony in 1993:

“A deeply personal, unique process of changing one’s attitudes, values, feelings, goals, skills and/or roles. It is a way of living a satisfying, hopeful, and contributing life even with limitations caused by the illness. Recovery involves the development of new meaning and purpose in one’s life as one grows beyond the catastrophic effects of mental illness. Recovery from mental illness involves much more than recovery from the illness itself.” [34] (p. 15).

The definition by Anthony describes properties of personal recovery and is focused on the personal and distinctive process of learning to live with continuing challenges and to be able to control difficulties as they surface [32]. In England, ideas about recovery have been “driven by professionals and policymakers and transformed from the journey of an individual to a model of service provision” [35] (p. 29). However, according to Perkins and colleagues [35] “as mental health services have taken ownership of recovery, its origins have been sought in the development of services rather than the journeys of those individuals whom they serve.” A complementary understanding of mental health recovery therefore focuses on “a contextual and social approach, focusing on social relationships, social roles and social inclusion. This orientation can be referred to as “social recovery”” [36] (p. 90–99).

Personal and social understanding of how people with mental health problems experience processes of recovery is vital in developing recovery-oriented mental health practices [16,37,38]. As Solli and colleagues state, “research on personal and social recovery gives primacy to idiographic knowledge”, remarking that people with a mental illness are ‘‘experts by experience and therefore in a position to provide valuable insight in what fosters and what hinders a good life for the individual person in a community” [16] (p. 247).

While the mental health recovery approach has gained momentum over the past decade and is providing standard models of mental health care in some parts of the world and while governments support recovery as a principle, implementation processes are still ongoing. A review of mental health recovery programs in various countries concluded that, although there seemed to be an acceptance of recovery being an important domain in health care, it is still a work in progress that requires sustained resources and commitment [39].

An underlying context for the development of the recovery perspective is that “general expectations of clinical recovery from severe mental illnesses have traditionally been pessimistic and progressive deterioration has mostly been the expected outcome” [32] (pp. i–ii). However, this perspective is challenged from two positions. Firstly, longitudinal studies showed that between one third and a half of people diagnosed with schizophrenia experience partial or full recovery [40,41,42]. Research also shows that “social interactions is a significant positive predictor of clinical recovery over a two-year period for persons in first-episode psychosis” [43] (p. 209). Furthermore, a meta-analysis of 92 studies documented that adults with major depression no longer meet criteria for major depression after treatment with psychotherapy [44], as symptoms including insomnia or hypersomnia, agitation, fatigue, feeling worthless, and thoughts of death or suicide had decreased. Secondly, research on personal experiences from people impacted by mental illness [45,46,47,48,49] have drawn attention to new perspectives on life quality for persons with ongoing mental health challenges. This body of knowledge has also helped to reclaim and redefine the term recovery. While clinical recovery refers to an observable full symptom remission [40], which means ‘getting back to normal’, mental health recovery refers to “recovering a life”, developing new meaning and purpose even within the limitations caused by illness [50] (p. 1).

## 3. Recovery and Music

Some of the developments in music activities resonate with perspectives of recovery. For example, advocates of community music have pronounced a contextual understanding of illness and health in response to individualized models of treatment [51]. Community music emphasizes active collaboration between individuals who play, create, improvise, and perform music together. Community music therapy illustrates some of the possibilities of music engagement in community settings as a means of social inclusion and participation [52].

Furthermore, focusing on empowering participants and the development of resource-oriented approaches to music therapy in mental healthcare [53,54,55,56] has stressed the promotion of positive health and building strength and emphasized qualities of autonomy and positive collaborations with others. Moreover, literature connected to the recovery perspective puts emphasis on empowerment and the service user engaging in music therapy [57,58,59]. Finally, related to contextual and resource-oriented approaches, there appears to be a growing understanding of the potentials of music activity having health promoting qualities that can be used in everyday life contexts [60,61,62]. Hence, a growing interdisciplinary field of research is concerned with music, health, and wellbeing [29,63,64].

However, only a few studies overtly link music therapy to recovery. Possibilities for enabling individuals to live satisfying lives in communities are discussed by Cynthia [63], who put emphasis on enabling empowerment, hope, and collaboration. Jensen & Allen [64] elaborate on how music therapy can promote social inclusion, collaboration, and responsibility in a community setting. Other authors [18,54] discuss the model of recovery in their demonstration of a contextual and resource-oriented approach to music therapy intended for people experiencing serious mental illness. Other studies have included in-depth introductions to recovery approaches in music therapy [5,15,65,66,67], including music recovery in a high security hospital context [68].

All of these studies indicate a connection between the therapeutic and wellbeing relevance of music and the determination of mental health recovery.

## 4. How Can Music Activities Support Recovery Processes?

Acknowledging that appreciation of music varies across individuals, research shows that there are positive similarities between accounts of service users’ experiences with music activities and recovery processes [16]. The similarities, as we understand it, are that service users describe their experiences with music as encounters that go further than the reduction of symptoms and clinical recovery from mental illness by opening up existential aspects that empower the individual’s life. From such perspectives, personal and social recovery places agency in the person living with mental illness [32,33]. The service users’ agency and involvement are therefore key components when exploring the possibilities of using music activities in recovery processes. Using the conceptual CHIME framework as a backdrop, we ask the question: How can music activities support recovery processes?

***Connectedness***—see Table 1.

### 4.1. Feelings of Equality between Service Users and Staff

A qualitative study shows [67] that songwriting may provide a platform for collaborative engagement which can help break down barriers between mental health service users and mental health staff. The study suggests that a songwriting process helped remove the perceived “hierarchical barriers” (p. 54) between service users and staff, and further enabled them to view each other from new perspectives. The service users also described “feelings of equality” as an agent, enabling them to participate within the song writing process. A service user remarked how the process made him “feel free” from being a service user. These findings align with other findings where service users describe experiencing “freedom” from illness and stigma within a music activities setting [18] (p. 67).

### 4.2. Social and Emotional Wellbeing

Many qualitative and quantitative studies have illustrated the benefits of choral singing for social and mental wellbeing [69,70,71]. Service users’ lived experiences suggest that group singing can act as coping strategies for those who are experiencing demanding life situations [72].

Connectedness was a key finding in the qualitative study of the user-led activity ‘Sing Your Heart Out’ (SYHO) project in Norfolk, UK, [73]. A community-based network of four singing workshops included individuals who had experienced mental ill health as well as the broader public. The project built on the therapeutic effects of singing, as well as singing with others. As a user-led activity promoting connectedness, control, hope and empowerment, the project drew on a recovery focus. All participants expressed “improvement” in, or “maintenance” of, their mental health wellbeing as a direct result of engagement in the singing workshops. For most of the participants this was a key component; singing and an inclusive social aspect was experienced as essential in the recovery process. Not feeling pressure to talk about illness and not engaging in a therapeutic process was also welcomed. Singing and being social was reported as promoting a feeling of belonging and wellbeing.

### 4.3. Tolerance

An evaluating study by McCaffrey and colleagues showed some personal challenges posed in music activities [15]. Participating mental health service users described many challenges such as relational challenges in group sessions where tolerance can be required. These findings that suggest that there are various social demands within a music group context were also acknowledged by Jampel [74].

***Hope and optimism***—see Table 2.

### 4.4. Hope and Social Agency—A Spiral of Positive Processes

In a meta-synthesis of service users’ experience, a combination of hope, joy, and social participation appears in numerous studies about music and mental health recovery [16]. As an example, a participant from a qualitative study [75] commented on how he found hope through music activity: ‘‘This experience of being able to contribute in a positive way has given me a stronger will not to give up in my crisis” (p. 308). Later, the same person joined a choir and started going to dance classes. The participant further commented: ‘‘I feel that when I go out in the world […] I enjoy it [the world] in a new way’’ (p. 319). This supports a spiral of positive experiences of joy, hope, agency, and social inclusion. While studies show the capacity of music to instill hope [2,3], create feelings of “wholeness” [76] and act as emotional expression [15,77], the area requires further research to better understand specific connections to the recovery process.

### 4.5. Triggering Encounters with Music

The concept of ‘musical recovery’ represents a process of regaining healthy relationships based on music promotion [15,16]. However, according to Bibb and colleagues [78], there are factors which can interfere both negatively and positively within a ‘musical recovery’ process. Research has identified ways that participation in making group music can evoke both negative emotions and memories [79], as well as promoting emotional distress for people in recovery [80]. Participants stated how triggering reactions can be helpful within a ‘safe’ context of a music therapy session. Thus, according to Bibb, triggering encounters with music do not necessarily mean a setback. Rather, these experiences can support the musical recovery process. Some of the participants from Bibb’s study reported that responses often depended on their fluctuating mental states. For example, a service user described how listening to music when feeling sad could keep him from self-harming behavior transferring his sadness “into the music”.

The relationship between music activities and recovery has also been identified in McFerran’s study [77], suggesting that the use of music depends on the mental state of being. This aligns with other studies illustrating how listening to music during periods of depression can enable people to transfer sad feelings into songs and how this can be important within the recovery process when individuals experience urges to self-harm [81].

The use of music to intensify or ruminate about existing thoughts and feelings has been explored by Garrido and Schubert [24] arguing that some people who experience difficulties in managing their mood listen to sad music. Participants in their study described the negative nature of music use and how music was used to reinforce existing pathology. This is done, even though little benefit is gained, because of difficulties in disengaging from negative emotional experiences. Based upon this, for many of the participants in the study, listening to music which corresponded with their mood exacerbated their mental state by creating feelings of hopelessness. These results demonstrate a need for a collaborative approach to practice [82] which supports users within their music experiences and pathways to recovery [83,84]. It also emphasizes the need for an initial facilitation when using music activities in recovery.

***Identity***—see Table 3.

### 4.6. Music Redefined and Reframed

“Understanding, redefining and accepting self; incorporating illness; and overcoming stigma are some of the essential building blocks of recovery” [85] (p. 113). Working with identity is essential in the recovery process [86,87]. It is a personal and a social process that has no endpoint [88] and the identity transformation can also be ongoing. The focus on identity is evident in McCaffrey’s qualitative study [5] where a service user stated that participating in musical activities offers an environment where people ”really can be themselves” (p. 130) promoting a positive self-image that built from feelings of achievement and success from participation. The freedom and achievement acquired within this process “redefine and reframe the limiting lens from which the participants viewed themselves in circumstances beyond musical activity” [89] (p. 45). A space where common artistic and human values are shared, developing agency and empowerment [90,91] is termed “musicking”. This process of play or, musicking, is experienced as a type of “drug” and for a service user it is a chance to “get out there and do my thing”. This is similar to other service users’ experience of music as a medicine or “legal dope” [92] (p. 77).

***Meaning***—see Table 4.

### 4.7. Recovery Understood as a Social Practice

Over a period of many years, mental health recovery has been understood as a unique and personal process [34], meaning that individuals need to ‘take control’ of their own lives [93]. In recent years, this is considered the core element of the recovery [94,95]. However, more recently, there has been an increased understanding that recovery is more than a personal process and that an individual has surfaced [36]. It is now also considered to be a social and relational process that occurs in the “everyday life” of individuals [96] (p. 130), in environments that are meaningful, in relations with other people, and by having access to activities and having valued social roles [36,97]. In this way, mental health recovery is about re-claiming a position as a contributing individual within their communities [38,98], with everyday experiences that may serve as important elements in the recovery process [96,99].

### 4.8. Moments of Flow and Peak Experiences

In a qualitative study, participants described moments of sharpened focus, and experiences of the outside world that “seemed to fade away and nothing other than being in a creative process seemed to matter” [100] (p. 10). A participant stated: “It is about focusing on the present moment. Here and now. It is about standing there, remembering lines, and not thinking” (p. 6). Participating in singing and playing activities was valued and described as an empowering experience that was useful in everyday life and beyond. For some of the participants, these moments lasted longer than the actual situation, and they were able to use the moments to both pursue creative work, and “as a reference point for life in general” (p. 11). The participants in the study also described moments when they had “exceptional experiences of joy or achievement” (p. 11); the participants entered a free zone, a place where their negative/sad thoughts or destructive behaviors received less attention [100]. Some of these moments may be seen as peak experiences (e.g., moments of focus or “flow experiences” of a world that seemed to fade away). Participants commented on how minor, positive moments had the potential to change aspects of their understandings of themselves and allow them to see their lives in another way. A participant described: “The sense of achievement and the joy that I felt that spring! It was one of those amazing experiences. To hold on to and enjoy each moment. It strengthened me so much …” (p. 7). The moments created hope, which led to them being more able to manage situations in a sometimes chaotic life. The participants’ encounters with music and theatre challenged beliefs about their potential for achievements as long-term mental health service users. Some participants stated that such moments were “guiding stars” (p. 11) in their lives that was central to their recovery. The flow and peak experiences had impacted positively on the participants mental health.

### 4.9. Moments of Meaning

“Participating in music and theatre workshops made lives of service users’ with long-term mental illness more meaningful” [100] (p. 7). A participant stated:

“Yeah, especially for the first few years, the music and theater workshop held me up. It was the only thing I could do except from lying in bed unable to do anything […] I remember it lifting me up, lifting me up from Wednesday to Wednesday, and then to more and more practices. Then I remember how I finally got out of the mud. It lifted me out of my depression through the hours I spent practicing every week”.

Participating in the music and theatre workshop offered the participants a central point in their everyday lives. This is coherent with findings from Borg and Davidson’s study [96], where participants stated that “even small reference points were of importance” to experience a “sense of meaning in everyday life” (p. 138). Attending a weekly rehearsal, or other meeting during the week, appeared to add to the participants’ coping skills. They got small glimpses of managing to hold on to an everyday routine by attending an activity. The authors of the music and theatre study [100] suggest that the participants described how they suddenly experienced meaning while performing—some for a few minutes, while others for longer intervals. Participants in the study emphasized the experience of meaning as a central positive component in a recovery process, showing consistency with previous studies [101,102] where findings showed that participatory arts activities can provide experiences of meaning.

***Empowerment***—see Table 5.

Having control over one’s life, focusing on strengths, and employing personal responsibility are three crucial aspects of personal recovery. According to Davidson and colleagues, it is important to be included in activities that are experienced as empowering and meaningful [48], even if symptoms increase for a while. It is, however, common in mental health care that service users have to wait until they are symptom free to be offered participation in activities.

### 4.10. Empowerment through Continuity in Cultural Activities

A study [103] focusing on mental health service user’s participation in a music workshop emphasized the importance of having continuous access to an activity even when hospitalized, to maintain routines and social engagements despite symptoms. The study indicates that, in order to ensure that people with long-term mental health problems participate, it is crucial to implement “activities that are flexible, person centered and resource oriented, where participants have participatory possibilities regardless of symptoms, functional ability, or whether they are hospitalized” (p. 1600). Furthermore, being offered creative growth and an illness-free zone to the participants in a hospital setting was considered important. The study (a music workshop) provided a space for both participants in hospitals and those in community care, providing continuous participation opportunities regardless of symptoms or limited functionality. In accordance with Borg and colleagues [104] and Solli [20], continuity in cultural activities is significant for individuals with long-term mental health problems. Moreover, flexibility is important, as people may live with their mental health illness [105] and there is a need for places that are inclusive of a spectrum of people at different stages of their recovery.

### 4.11. Potentials Instead of Limitations

A qualitative study found that completing and managing challenging tasks created a possibility for creative growth. The participants were considered to be musicians and actors, rather than people living with mental health illnesses—even if the material in the workshops (songs, poetry, and writing scripts) was based on narratives from living with mental health problems.

According to Ørjasæter and colleagues [103], interacting with art professionals who would see potential instead of limitations helped the participants access opportunities. To Ørjasæter, looking at symptoms in other ways might lead to service users finding strategies to live with their symptoms and to accept them. During the study, the participants’ challenging problems minimized, as they did not have to hide their symptoms or use so much of their cognitive capacity on the symptoms’ effects. In a creative setting there might be more tolerance of diversity, and the idea of “madness” is more acceptable than in traditional health settings (p. 1609). Similarly, based on a qualitative study, Lloyd and colleagues argue that participation in cultural activities can promote a lifestyle where participants can identify themselves aside from their mental health challenges [106].

Slade [32] characterizes the mental health system’s tendency to use “dichotomous scales to explore people in terms of normal–abnormal, sick–healthy, us–them, and patients as nonexperts–health professionals as experts, resulting in both health professionals and people with long-term mental health problems may internalize a ‘narrow’ normality” (pp. i–ii). Slade considers it as “a paradox”, that a system which is theoretically there to help people has narrow limits regarding what is normal [32].

## 5. Discussion. How Can Music Activities Contribute to Recovery-Oriented Practice and Services in Mental Health Care?

### An Overview

Within the structure of the conceptual framework CHIME, we have identified important aspects and key components of music activities in recovery processes based on perspectives of mental health service users—see Table 6.

We now discuss the findings, inspired by McCaffrey and colleagues’ four central ways of empowering the contribution to recovery-oriented practice and services in mental health care [77]. The four central ways of empowering mental health practice are identified as: expertise by experiences, personally defined recovery, personal autonomy, and social participation.

First, it is crucial to recognize and respect *expertise by experience*. It is all-important to regard service users as experts because of their experience. An important understanding in mental health recovery is that service users gain expertise by living with mental illness [107]. This perspective places service users as equal partners in the treatment process, where personal experience encounters professional expertise that is gained by skill and/or training. This might inform professionals of service users’ unique knowledge in understanding aspects that may foster a personally meaningful and fulfilling life as well as their own priorities [77]. This further fosters an openness to the service user as all-important when implementing music activities as recovery-oriented practice. Seen from an organizational perspective, the practical implications of recognizing service users’ expert role would include involving service users and user organizations in the development of participatory activities, such as music engagement. Another possibility is to include service users or people with user-experience and their relatives as partners or peer-mentors.

Second, recovery-oriented practice acknowledges *personally defined recovery*, where “individuals are supported to define their own needs, goals, dreams, and plans for the future to shape the content of care” [108] (p. 1474), rather than by generalized activities founded on diagnosis and function. Hence, elements that further recovery and music activities should be determined by the individual’s own preferences.

Third, recovery emphasizes the individual’s *personal autonomy* and strengths and it is suggested that the overall aims of therapy support service users’ resources, rather than focusing on weaknesses as seen within a medical model of practice [95,109]. However, it is important to state that the starting point on the person’s resources does not mean the avoidance of challenges and illness. Rather, in line with Rolvsjord [110] there is a need for a better balance between the focus on resources and challenges, as both are intertwined in the mental health recovery process.

The connection between music engagement and mental health is still unclear [111]. Therefore, it is imperative that a form of ‘guidance’ be available when music is used as an activity in recovery-processes. However, we suggest that an increased focus on wellbeing and positive aspects of mental health can be found through engaging in music activities, as many service users are challenged with stigma, hopelessness and low motivation [112].

Fourth and finally, as “people with mental health problems often experience stigma, disempowerment, and social exclusion, processes of recovery are interlinked with social processes of change and a need for *social participation*” [113] (p. 31). Therefore, an important focus within recovery-oriented services is to help people who live with mental illness to join activities in their communities as equal citizens [108]. From this perspective, music activities have proven to be a supportive platform for developing positive relationships, building social networks, and facilitating the transition from a hospital setting to living at home [60,114]. We suggest that to encourage the use of music activities in recovery there needs to be focus on elements of social participation and inclusion. Social activities and preservation of community links should be promoted as much as possible. This can include taking part in music activities within institutions or in the community such as music making in choirs or bands, singing or writing songs [74,83].

There are, however, and according to Slade, structural challenging conditions for promoting personal and social recovery [32]. Standardized guidelines and manuals are in focus, and effectiveness and outcomes of treatment are constantly measured. The application of a recovery-oriented approach, where the service user guides the process and the goals, has proven to be difficult, according to Slade [30]. Focusing on issues of empowerment in recovery is an important way of meeting these challenges. Therefore, in agreement with McCaffrey [77], we argue that developing and facilitating engagement in music activities, with a focus on strengths and resources in the individual, promoting community inclusion, as well as humanistic values, is a valuable way of promoting ‘creative’, recovery-oriented approaches.

## 6. Conclusions

This essay has presented literature exploring how music activities can support mental health and recovery processes, seen from service users’ perspectives. Engagement in music activities has the potential to promote interpersonal communication. Music can initiate a spiral of positive processes such as feelings of hope and being connected that can promote personal and social agency, wellbeing, and mental health recovery. We propose that to maximize support for recovery, a focus on social participation, inclusion, and participation in music activities can help develop positive relationships with others, expand social networks, support personal recovery, and strengthen social participation as well as create positive, joyful experiences.

## Figures and Tables

**Table 1:** Sub-components of the CHIME recovery framework [27].

Connectedness “Having good relationships and being connected to other people in positive ways. Characterized by peer support and support groups; support from others; community” [Recoveryplace: https://www.therecoveryplace.co.uk/chime-framework/]

**Table 2:** Sub-components of the CHIME recovery framework [27].

Hope “Having hope and optimism that recovery is possible and relationships that support this. Characterized by motivation to change; positive thinking and valuing success; having dreams and aspirations” [Recoveryplace: https://www.therecoveryplace.co.uk/chime-framework/]

**Table 3:** Sub-components of the CHIME recovery framework [27].

Identity “Regaining a positive sense of self and identity and overcoming stigma” [Recoveryplace: https://www.therecoveryplace.co.uk/chime-framework/]

**Table 4:** Sub-components of the CHIME recovery framework [27].

Meaning “Living a meaningful and purposeful life, as defined by the person (not others). Characterized by meaning in mental ‘illness experience’; spirituality; meaningful life and social roles and goals, rebuilding life” [Recoveryplace: https://www.therecoveryplace.co.uk/chime-framework/]

**Table 5:** Sub-components of the CHIME recovery framework [27].

Empowerment “Having control over life, focusing on strengths, and taking personal responsibility” [Recoveryplace: https://www.therecoveryplace.co.uk/chime-framework/]

**Table 6:** Key components of music activities in recovery processes based on perspectives of mental health service users.

CHIME	Key Components of Music Activities in Recovery Processes Based on Perspectives of Mental Health Service Users
	
Connectedness	- Feelings of equality between service users and staff - Social and emotional wellbeing - Tolerance
	
Hope & optimism	- Hope and social agency—a spiral of positive processes - Triggering encounters with music
	
Identity	- Music redefined and reframed
	
Meaning	- Recovery understood as a social practice - Moments of flow and peak experiences - Moments of meaning
	
Empowerment	- Empowerment through continuity in cultural activities - Potentials instead of limitations

## Data Availability

No new data were created or analyzed in this study. Data sharing is not applicable to this article.
